# Hunter–gatherer mobility and technological landscapes in southernmost South America: a statistical learning approach

**DOI:** 10.1098/rsos.180906

**Published:** 2018-10-10

**Authors:** Ivan Briz i Godino, Virginia Ahedo, Myrian Álvarez, Nélida Pal, Lucas Turnes, José Ignacio Santos, Débora Zurro, Jorge Caro, José Manuel Galán

**Affiliations:** 1 CONICET-Centro Austral de Investigaciones Científicas, B. Houssay 200, Ushuaia 9410, Argentina; 2 Department of Archaeology, University of York, The King's Manor, Y01 7EP York, UK; 3 Departamento de Ingeniería Civil, Universidad de Burgos, Edif. A1. Avenida Cantabria, S/N, 09006 Burgos, Spain; 4 Departamento de Ingeniería Civil, INSISOC, Universidad de Burgos, Edif. ‘La Milanera’, C/Villadiego, S/N, 09001 Burgos, Spain; 5 Cultural and Socio-Ecological Dynamics Research Group (CaSEs) – GRPRE 212, Barcelona, Spain; 6 Department of Archaeology and Anthropology, IMF-CSIC (Spanish National Research Council), Carrer de les Egipcíaques 15, 08001 Barcelona, Spain; 7 Department of Humanities, Universitat Pompeu Fabra (UPF), Jaume I Building (Ciutadella Campus), Carrer de Ramon Trias Fargas 25-27, 08005 Barcelona, Spain; 8 Department of Sociology, GSADI, Autonomous University of Barcelona (UAB), Campus UAB, 08193 Cerdanyola del Vallès, Spain

**Keywords:** hunter–gatherer, statistical learning, shared technology, random forest, mobility

## Abstract

The present work aims to quantitatively explore and understand the relationship between mobility types (nautical versus pedestrian), specific technological traits and shared technological knowledge in pedestrian hunter–gatherer and nautical hunter–fisher–gatherer societies from the southernmost portion of South America. To that end, advanced statistical learning techniques are used: state-of-the-art classification algorithms and variable importance analyses. Results show a strong relationship between technological knowledge, traits and mobility types. Occupations can be accurately classified into nautical and pedestrian due to the existence of a non-trivial pattern between mobility and a relatively small fraction of variables from some specific technological categories. Cases where the best-fitted classification algorithm fails to generalize are found significantly interesting. These instances can unveil lack of information, not enough entries in the training set, singular features or ambiguity, the latter case being a possible indicator of the interaction between nautical and pedestrian societies.

## 1. Introduction

The identification of human groups, social boundaries and interactions based on material culture lies at the heart of archaeological research. Different methodological approaches have been applied to a broad range of case studies, focusing on the diverse dimensions of social materiality [1–3]. However, the selection of reliable archaeological markers to tackle these issues remains a matter of debate.

Coastal settlements offer stimulating case studies to identify distinct groups and to explore connectedness. Exploitation of marine resources provided the scenario for changes in socio-economic organization, as well as for the emergence of new technologies and novel forms of interaction [4–8]. Among these technological innovations, seafaring was undoubtedly an evolutionary key element for hunter–fisher–gatherer (HFG) populations. Sailing technology not only increased their mobility range allowing the colonization of new territories and biotopes, broadening socio-economic practices [9,10] but also enhanced social connectedness, reinforcing social identities [11] and creating new social landscapes.

Hence, several authors emphasized the relevance of distinguishing between nautical and pedestrian populations who inhabited the coasts [9,12,13]. However, from the point of view of HFG archaeology, this distinction remains problematic. Coastal HFG sites are generally classified as nautical or pedestrian based on the exploitation of offshore resources or the appearance of human presence in oceanic islands [5,14]. Nonetheless, it is important to be careful when classifying HFG sites as nautical based on the sole presence of intertidal resources, as it has been demonstrated that it is not a direct proof of the existence of seafaring [9].

Despite the interest in distinguishing between coastal societies with and without seafaring technology, no formal assessment considering material culture patterns has been conducted yet. Even though connections between resource procurement and specificity of technologies point to a significant relationship, so far, no formal quantitative evidence confirms the strength of this relationship, neither at individual nor at the group level (mesoscale analysis). The general global pattern has not been assessed either. Therefore, the development of analytical procedures to disentangle coastal–inland dynamics in hunter–gatherer (HG) and HFG societies has a significant methodological value in the archaeological field [15].

In this work, we selected a novel approach based on advanced statistical learning techniques and applied it to HG and HFG societies who inhabited the uttermost part of South America during the Holocene, with the aim of identifying the technological variables that better discriminate between nautical and pedestrian populations. Therefore, in the present case study, the term ‘mobility’ refers to the distinction between nautical and pedestrian mobility types.

The archaeological research conducted in this area pointed to the presence of nautical and pedestrian societies, according to the aforementioned proxies for seafaring technology. Moreover, ethnographic observations recorded by European explorers during the nineteenth and the early twentieth century confirm the existence of maritime and terrestrial HG and HFG societies in the Fuegian Archipelago [16,17], including evidence of social interaction and information flow between those populations. Nevertheless, it is worth noting that prior analyses have focused on the geographical distribution of particular items of material culture to trace contacts. Examples of such items are raw materials [18–20], decoration patterns of portable art [21] or singular lithic and bone designs [22]. By contrast, in the present research, we rely on a comprehensive database which encompasses a wide range of technological variables to overcome the fragmentary nature of the archaeological record, thus broadening the analytical capabilities offered by material culture.

The main aim of this work is to explore the relationships between specific technological traits and different mobility and transportation modalities in HG and HFG societies, as well as to unveil the degree of connectedness between nautical and pedestrian communities. The spatial analysis of technological variables in nautical and pedestrian sites allows us to identify technological landscapes [23], a term that refers to the geographical distribution of technical traits or practices, including uses, designs and knowledge, in a specific time-frame [23,24]. Technological landscapes imply a particular social context of shared technological knowledge and may allow us to define areas of interaction.

The set of preliminary assumptions that guide the exploratory analyses conducted may be summarized as follows: (i) the patterning of material culture is a direct result of the social relationships between individuals and groups in which these objects were produced, used and circulated [25]; (ii) different mobility capabilities are related to different technological developments; (iii) a set of technological variables may be the most discriminant in order to distinguish between nautical and pedestrian mobility; (iv) sites exhibiting technological features characteristic from both mobility types may indicate interaction areas between nautical and pedestrian populations.

The first approach selected to deeper delve into the possible relationships between mobility and technology consists of implementing different supervised learning classification algorithms. Each classifier uses a different set of assumptions—inductive bias—to generalize beyond the data. To choose the appropriate one, improve accuracy and capture the underlying association patterns between variables, we have compared through stratified 10-fold nested cross-validation [26,27] several benchmark and top classification algorithms. These classification algorithms detect on our data significant patterns between mobility types and certain technological elements, traits or knowledges. Afterwards, with the aim of identifying the variables that better discriminate between both mobility types, individual and group variable importance analyses were conducted by implementing diverse state-of-the-art variable importance analysis methodologies.

## 2. Material and methods

### 2.1. Data

To understand the potential relationship between technology and nautical and pedestrian mobility capabilities, we compiled all the available information (more than 250 publications, official reports and manuscripts) about 201 archaeological sites from Southern Patagonia, collecting spatial, chronological and technological data [28]. This large database includes 258 occupations ranging from the Pleistocene-Holocene transition (13 000 BP), following [29] to the European colonization in the final period of the nineteenth century [30–32] involving inland and coastal archaeological sites. Within the database, 52.71% of the occupations (*n* = 136) are sites identified as pedestrian by the archaeological literature, i.e. corresponding to HG and HFG groups with pedestrian mobility, while the rest of the occupations (*n* = 122) are identified as nautical, i.e. corresponding to HFG groups with nautical technology.

To increase the sample size, assemblages retrieved from undated deposits were also included in the database. Given the information gathered, ranging from archaeological research conducted in the study area from the 1930s [33] until today, terminological differences had to be overcome to ensure a homogeneous and operational database. All data collected from the papers, books and manuscripts consulted was critically reviewed and homogenized under unified criteria. The final database includes technological information about lithics, bone tools and artefacts on the shell, considering raw materials, manufacture techniques, design and decoration. In terms of variables, it comprises 187 variables and frequency data. For the purpose of the present research, the database was normalized into a binary matrix (presence/absence).

**Figure 1.**
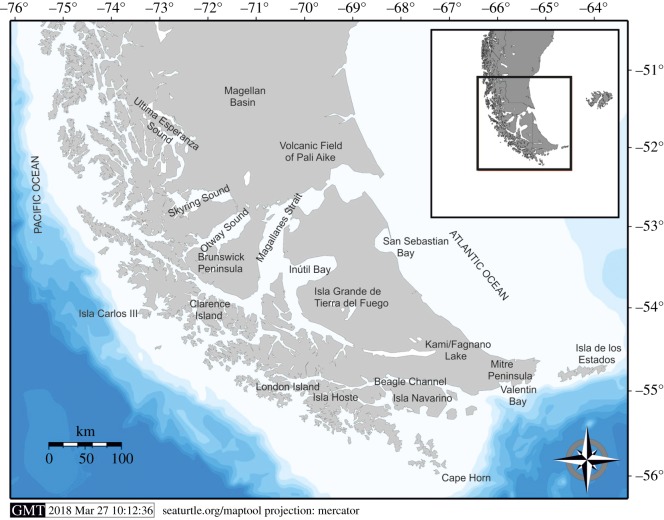
General map of the study area.

The database is focused on the archaeological record of the southernmost portion of South America: from 51°S until Cape Horn (55°S), involving inland and coastal environments. The study area includes in the continent, the Austral Magellan Basin, the Andean area, the Pacific and Atlantic Coasts and the Patagonian Steppe (figure 1). The insular context encompasses the Fuegian Archipelago: a pre-eminent marine environment formed by a mainland mass, Isla Grande de Tierra del Fuego (IGTDF), surrounded by numerous and minor islands, including Isla de los Estados and Isla Hornos. The selected area is suitable for the aim of the present study, since it involves different landscapes where, according to the geographical localization of the archaeological sites, pedestrian and nautical mobility capabilities can be expected, either inland or near to maritime or lacustrine shorelines.

### 2.2. Classification techniques

Classification is the problem of assigning a new observation to a class among a set of categories or labels. In statistical and machine learning, this assignment is based on the pattern learned from a training set of observations whose label is known (supervised learning). In our case study, we are interested in learning to differentiate between occupations with nautical or pedestrian mobility patterns (labels), based on archaeological technological evidence.

In classification, there is no algorithm that outperforms all the others in all possible cases [34]; consequently, it is necessary to select the most appropriate classifier for each context. According to recent research, some of the most general cutting-edge classification algorithms include random forest, rotation forest, boosting and support vector machines (SVM) among others [35,36].

In addition to these classification algorithms, other benchmark classifiers have been implemented to establish a baseline and to check if patterns between the inputs and the output are significant or not.

The set of algorithms considered is the following: random forest, rotation forest (J48 as base learner), AdaBoost (J48 as base learner), SVM using Gaussian and polynomial kernels, J48 decision tree, naive Bayes, OneR and ZeroR. Then, the different results were compared, and the model that best fits our data was selected by 10-fold nested cross-validation.

*Random Forest* is based on the construction of different classification trees on bootstrapped samples of the training set. Apart from bagging (bootstrap aggregation), random forest includes the random subspace method, which consists in forcing each split to consider only a subset of predictors when building a tree; this mechanism helps to decorrelate the different trees built for the same forest [37].

*Rotation Forest* was first conceived with the aim of building accurate and diverse classifiers (bias-variance trade-off) [38]. To create the training data for each base classifier, the feature set is randomly split into *K* subsets; then, for every such subset, a non-empty subset of classes is randomly selected and a bootstrap sample of size 75% of the data count is drawn. Next, principal component analysis is run. In the end, a full feature set is reconstructed to build each classifier in the ensemble. Eventually, each item is assigned to a class according to the majority vote calculated over all the trees in the ensemble.

*AdaBoost* [39] was the first practical boosting algorithm. However, it remains one of the most widely used, with applications in numerous fields. Boosting aims at creating a highly accurate classifier by combining weak classifiers. In contrast to bagging, which fits a separate decision tree to each bootstrapped copy of the dataset and combines all the trees to create a single predictive model, in the case of boosting, trees are grown sequentially, each tree being grown using information from previously grown trees. Instead of implementing bootstrap sampling, in boosting algorithms, each tree is fit on a modified version of the original dataset, i.e. the residuals of the previous model, so that the new tree performs better on the points where the previous one performed poorly.

*J48* is an open source Java implementation of the C4.5 algorithm in Weka. C4.5 is an algorithm used to generate a decision tree developed by Ross Quinlan [40]. C4.5 splitting criterion is the normalized Information gain (IG) (difference in entropy). The attribute with the highest normalized IG is chosen to make the decision.

*SVMs* are an extension of the support vector classifier that results from enlarging the feature space using kernels [41,42]. The support vector classifier is based on the construction of a hyperplane such that it correctly separates most of the training observations into two classes. Then, an observation is classified depending on which side of the hyperplane it lies. It is a perfectly valid approach if the boundary between the two classes is linear. Unfortunately, linear boundaries are usually not the case, and that is why in SVMs the feature space is enlarged to accommodate a nonlinear boundary between the classes. The kernel approach is simply an efficient computational technique for enacting this idea. Several kernels exist and none has proved better than the rest. Therefore, the selection of the kernel type depends on the particular case. In the present work, two different kernels were tried: polynomial kernel and Gaussian kernel, the latter performing better on our data.

*Naive Bayes* or Bayes classifier is a very simple probabilistic classifier which is also quite popular due to its good performance in many applications. It assigns each observation to the most likely class, given its predictor values [42]. This classifier relies on Bayes' theorem and assumes independence between the features, ignoring any possible correlations between them (naive assumption).

The last two algorithms, i.e. OneR and ZeroR, are characterized by their lack of predictive power; however, they are useful to establish a baseline performance (benchmark) for other classification methods.

*ZeroR* is a classification method which relies on the output and ignores all predictors. ZeroR classifier simply predicts the most frequent class, regardless of the predictor values, giving a lower limit on the accuracy [43].

*OneR* (One Rule) is a simple classification algorithm that generates one rule for each predictor in the dataset. At a later stage, it selects the rule with the smallest total error and establishes this rule as its ‘one rule’. In some cases, it has been shown that OneR produces rules only slightly less accurate than state-of-the-art classification algorithms, while producing rules by far simpler to interpret [44]. Therefore, OneR also provides a baseline performance.

### 2.3. Variable importance analysis and group variable importance analysis

Variable importance analysis can be performed both at individual and at aggregated level. In the present work, both analyses have been conducted. Variable importance analysis at the individual level has been computed using two main types of measures: model-independent measures (phi coefficient and IG) and a model-dependent measure (mean decrease in accuracy).

#### 2.3.1. Phi coefficient

The phi coefficient is a measure of association between two binary variables. It is similar to Pearson correlation coefficient in its interpretation. In fact, a Pearson correlation coefficient estimated for two binary variables will return the phi coefficient. It ranges from −1 to 1, where 1 stands for strong positive correlation, −1 for strong negative correlation and 0 for no correlation.

#### 2.3.2. Information gain

IG quantifies the amount of information that an attribute gives about the class using the reduction in the Shannon entropy.

2.1 $$\mathrm{IG}=H(\mathrm{class})-H\left(\frac{\mathrm{class}}{\mathrm{attribute}}\right).$$

Entropy is a magnitude that tries to measure how mixed data is with respect to a target variable (i.e. the class to which it belongs in the classification problem). Thus, if all classes are equally represented (maximal mixture), then entropy is maximal and vice versa. Features that are unrelated to the output produce no information gain, while features that perfectly partition should give maximal information.

#### 2.3.3. Mean decrease in accuracy

Mean decrease in accuracy is a variable importance measure for ensemble models (such as random forests), based on bootstrapping. The method takes the fitted model, performs a permutation test [37] and checks its impact on the prediction of the out-of-bag observations from a single bootstrapped sample [42]. If the variable under consideration is not important (null hypothesis), then rearranging the values of that variable will not degrade the prediction accuracy and vice versa. Therefore, one can evaluate the importance of a variable by quantifying the change in predictive accuracy after the permutation with respect to the initial case.

### 2.4. Group variable importance

In many situations, groups of variables can be naturally identified, and it is of interest to select groups of variables rather than to analyse them individually [45]. Permutation importance has been recently extended to groups of variables and specifically defined for the particular case of random forests [46].

Let *J* = (*j*_1_, … , *j_k_*) be a *k*-tuple of increasing indices in {1, … , *p*}, with *k* ≤ *p*. The permutation importance of sub-vector *X_J_* = (*X_j_*_1_, *X_j_*_2_, … , *X_jk_*)*^T^* of predictors can be defined as follows:

2.2 $$I(X_{J})=E[{\left(Y-f(X_{\left(J\right)})\right)}^{2}]-E[{\left(Y-f(X)\right)}^{2}],$$

where $X_{\left(J\right)}={\left(X_{1},\cdots ,{X^{\prime }}_{\;j1},X_{\;j1+1},\cdots ,{X^{\prime }}_{\;j2},X_{\;j2+1},\cdots ,X_{p}\right)}^{T}$ is a random vector such that $X_{J}^{\prime }={\left({X^{\prime }}_{\;j1},{X^{\prime }}_{\;j2},\cdots ,{X^{\prime }}_{\;jk}\right)}^{T}$is an independent replicate of *X_J_* which is also independent of *Y* and all other predictors. It is important to note that the same random permutation is used for each variable *X_j_* of the group. In this way, the (empirical) joint distribution of *X_J_* is left unchanged by the permutation, whereas the link between *X_J_*, *Y* and the other predictors is broken.

A rescaled measure (2.3) penalizing the group size can also be defined [46]:

2.3 $$I_{\mathrm{nor}}(X_{J})=\frac{1}{\left|J\right|}I(X_{J}).$$

## 3. Results

The analyses conducted in this research yielded three main results: (i) the recognition of the technological sets of nautical and pedestrian groups, (ii) the identification of technological landscapes and (iii) the assessment of social interaction patterns between both types of societies.

### 3.1. Technological sets of nautical and pedestrian groups

#### 3.1.1. Classification accuracy, ANOVA and post-ANOVA

For the present case study, random forest is the technique that best fits our data, with an average accuracy of 86.4%. In the first two columns of table 1, the average accuracy and the standard error of the classifiers are shown. Results were obtained using stratified 10-fold nested cross-validation. Stratification was chosen to ensure that each fold is a good representative of the whole, i.e. that the proportion of classes in each fold is the same as the proportion in the whole dataset.

**Table 1.** Average accuracy and s.e. of each classifier. Results were obtained using stratified 10-fold nested cross-validation. The test of equality of means can be rejected at the level of significance of 0.001 using an ANOVA test. Duncan's multiple range test for accuracy (alpha: 0.05) was used for *post hoc* analysis. Two classifiers are considered statistically different if the accuracy difference exceeds a studentized range statistic. Differences between classifiers that share a letter in the subgroup are not considered statistically significant.

classification method	average accuracy	s.e.	subgroup
random forest	86.446	2.396	A
SVM-norm. polynomial kernel	86.046	3.377	A
rotation forest (J48 as base learner)	84.908	2.263	Ab
AdaBoost (J48 as base learner)	82.584	3.162	Ab
SVM-Gaussian kernel	82.584	3.162	Ab
J48 decision tree	77.200	2.351	B
Naive Bayes	68.523	2.912	C
OneR	59.246	2.742	D
ZeroR	52.708	0.510	D

To assess the robustness of the classification techniques implemented, ANOVA and post-ANOVA tests were undertaken. The ANOVA test was conducted to check the null hypothesis of equality of means across all the algorithms implemented. The result is that the null hypothesis can be rejected at a level of significance of 0.001, which means that the more sophisticated algorithms perform significantly better on our dataset than the baseline ones. This implies that some patterns exist between the regressors (technology) and the output (mobility), rotation forest being the technique that best captures those patterns.

To complete the study, a *post hoc* analysis was conducted by implementing Duncan's multiple range test for accuracy, with a 0.05 level of significance (table 1). According to Duncan's multiple range test, two classifiers are considered statistically different if the accuracy difference exceeds a studentized range statistic. Each classifier is given letters according to the accuracy differences. Differences between classifiers with different letters are considered statistically significant while differences between classifiers that share a letter are not considered statistically significant.

Since the group variable importance analysis proposed is based on a permutation test conducted on a random forest fitted to the dataset, the fact that the model necessary to perform the analysis is the one with the highest accuracy on our data, reinforces the robustness and coherence of the global analysis.

#### 3.1.2. Individual variable importance

The results obtained from the individual variable importance analyses performed with both model-independent and model-dependent measures are coherent. The rankings obtained with each metric are slightly different from each other, but in overall terms, it can be asserted that the great majority of variables happen to be unimportant to discriminate between nautical and pedestrian mobility.

To analyse more in detail the few variables that are discriminant, the variables in the top 20 of the three metrics were considered. In particular, table 2 shows the 19 most important variables, arranged according to the number of metrics in which those variables are part of the top 20: 3 metrics out of 3 and 2 metrics out of 3.

**Table 2.** Ranking of the 19 most discriminant variables for mobility patterns according to the individual variable importance analyses. Here, we show the variables which are discriminant according to 3 out of 3 and 2 out of 3 metrics. The grey-shadowed variables are the variables that point to pedestrian mobility. The variables in white point to nautical mobility.

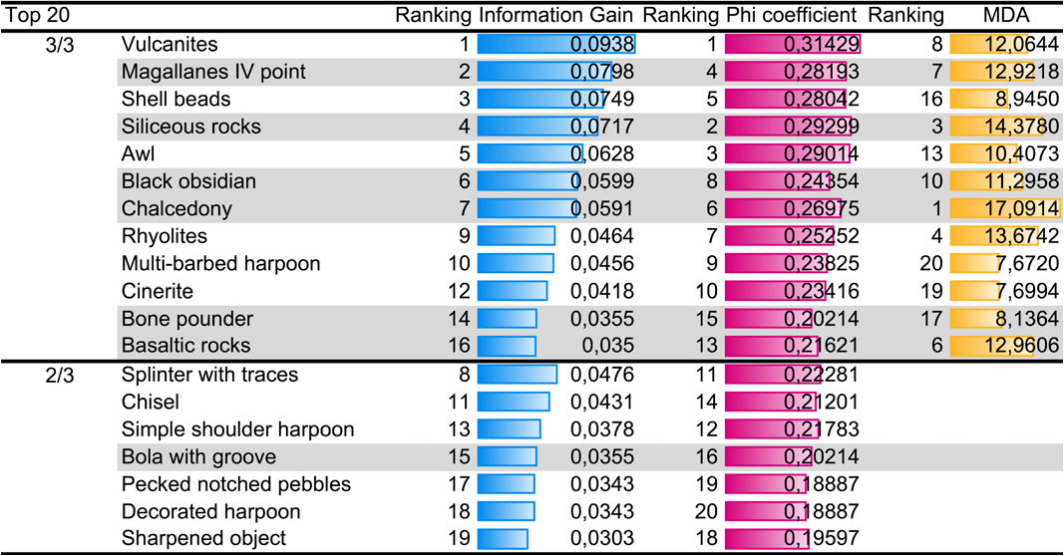

According to the results presented in table 2, the key discriminant variables include technological features related to some specific lithic and bone tool designs, together with lithic raw materials. Important diagnostic features for pedestrians are the projectile point known in the classical archaeology of southern Patagonia as ‘Magallanes IV’ [33], bone pounders and bolas with grooves. Nonetheless, it is necessary to clarify that in the archaeological record, the last two designs also appear in two different nautical sites of the Magellan Strait area.

Conversely, shell beads, a series of bone tools and pecked notched pebbles are salient technological characteristics of nautical societies. Shell beads, multi-barbed and decorated harpoons, splinters and chisels are limited to nautical occupations. On the contrary, awls, simple shoulder harpoons and sharpened objects were also used by pedestrian groups.

With regard to raw materials, the number of variables involved in their exploitation, such as geographical distribution, accessibility, the problems related to source identification and the biases related to the classification criteria used by different analysts, make it difficult to provide straightforward explanations. The procurement of rocks at available local sources nearby the archaeological sites partially explained the strong association between raw materials and mobility. This applies to basalts, chalcedonies and siliceous rocks linked to pedestrian mobility, as well as to rhyolites, vulcanites and cinerites associated with nautical groups. However, most of these raw materials are found in both groups because they present a relative ubiquitous distribution in secondary deposits, having been transported by the action of glacial and glaciofluvial processes during the Quaternary. Consequently, the presence of these rocks in the archaeological record does not necessarily imply inter-group connectedness.

The opposite is the case of black obsidian, whose geological region of provenance has been identified [47]; it is located in Pampa del Asador, around 47°50′17″ S and 70°59′11″ W, circa 400 km away to the north from the study area. This type of obsidian, exclusively associated with pedestrian societies, is distributed along the occupations of the continent but it also appears in Marazzi I, a site located in the IGTDF steppe (more than 600 km away from the source) after the opening of the Magellan Strait. Contrastingly, the other two raw materials available in primary sources in the study area, green obsidian and Miraflores rocks, follow a dissimilar pattern and exhibit a relatively wide distribution between nautical and terrestrial groups [20,48]. The source of green obsidian is located around Otway Sound, and the source of Miraflores rocks (tuffs and silicified tuffs) is situated in the Chorrillo Valley in the north of IGTDF. As the vast majority of the territory in Otway Sound was occupied by maritime societies, it has been suggested that green obsidian was distributed and controlled by these groups. On the contrary, terrestrial hunter–gatherers controlled the circulation of Miraflores rocks throughout IGTDF [19].

#### 3.1.3. Group variable importance

**Figure 2.**
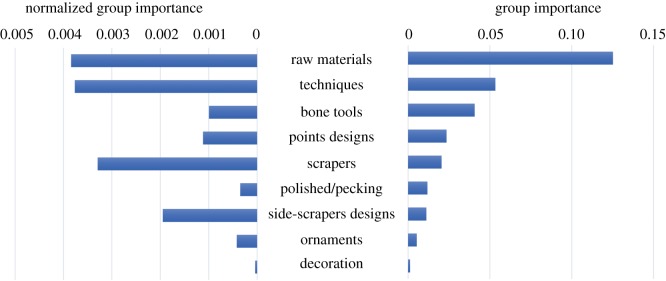
Group importance and normalized group importance of the nine groups of variables used as regressors.

Nine main aggregated categories have been considered because the 187 predictive variables can be accurately partitioned into them: raw materials, techniques, bone tools, point designs, scrapers, polished/pecking, side-scrapers design, ornaments and decoration. These nine groups happen to be of interest, mainly because of their synthesis capability. The results obtained can be seen in figure 2.

Two different group variable importance analyses were conducted: one without normalization of the variables and the other in accordance with the normalization procedure by Gregorutti *et al*. [46], which divides by group size. Since the most suitable normalization procedure for group variable importance analysis is still to be determined, the values obtained with Gregorutti's normalization are just presented as complementary information. It is the ranking given by the group variable importance analysis without normalization that is studied and analysed in detail.

Putting aside raw materials (because of the problems mentioned above related to their distribution and source identification), several interesting archaeological results arise from the analysis of the results in figure 2:

First, the group of techniques, which comprises different ways in which force was applied to produce an object, is more diagnostic than artefact morphologies. Second, bone tools and projectile points remain important. Third, in contrast to individual variable importance analysis, group variable importance points to scrapers and side-scraper designs as having a non-negligible significance to distinguish between mobility types. Fourth, interestingly, ornaments and decoration, which are commonly used in archaeology as diagnostic traits for identifying groups, appear at the end of the ranking, being consequently the least significant groups to distinguish between nautical and pedestrian mobility types. At this point, it is important to note that decorated items exhibit lower frequencies in the archaeological record in comparison to the rest of technological variables, which may have some kind of influence in the results obtained.

## 4. Discussion

### 4.1. Technological landscapes

**Figure 3.**
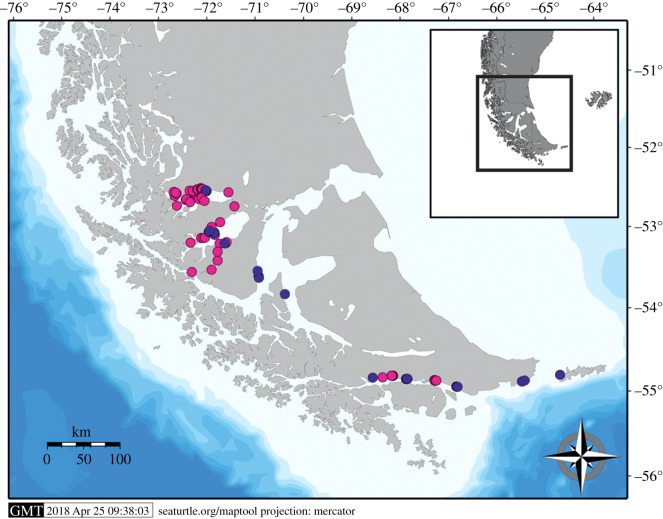
Map with nautical occupations. Blue dots are misclassified by the algorithm, i.e. the classifier predicts that the blue dots are pedestrian occupations, while in reality they are nautical; red dots are classified in coherence with the archaeological literature.

In archaeological research, the traditional procedure for identifying technological interaction consists of the analysis of the spatial connectedness of technological variables. By contrast, in the present study, the technological landscapes have been obtained by geo-positioning the sites classified as nautical or pedestrian by the random forest algorithm and by checking the classification obtained against the archaeological literature. This new methodology, aside from the representation of technological landscapes, enables the identification of the boundaries between them and, more importantly, the detection and analysis of conflictive points, possible indicators of interaction between groups. The geographical distribution of the sites classified as nautical by the random forest, encompasses a technological landscape which involves two areas: the coastlines of Otway-Skyring Sounds, and the Beagle Channel-Valentín Bay area (figure 3). Some sites included in this technological landscape are placed in the Westernmost section of Magallanes Strait, including Port Famine and the vicinity of Isla Carlos III. The absence of archaeological sites in the South-Western Channels (Clarence Island, London Island and IGTDF, among others) can be easily explained by the absence of archaeological research in this area.

This space of shared technology is not merely linked to marine ecosystems; it is related to a landscape of channels, islands and watercourses where seafaring technology was indispensable. In addition, it is interesting to note that there is no evidence of the use of nautical technology in HG societies of the Atlantic coast of IGTDF and Continental Patagonia: the relationship between seafaring and technological variability is strongly related to the Pacific Coast. One of the most relevant results obtained is that the geographical pattern of the nautical technological landscape supports the hypothesis of the arrival of nautical societies to Magellan–Fuegian Archipelago, in the Middle Holocene, through the Western Channels and Islands of the Pacific Ocean [49,50].

In the case of sites classified as pedestrian by the random forest algorithm, the technological landscape obtained presents a geographical distribution which is broader and more homogeneous than the nautical one, covering both inland and coastal areas. Some different concentration areas, with different sizes are present in the study area.

**Figure 4.**
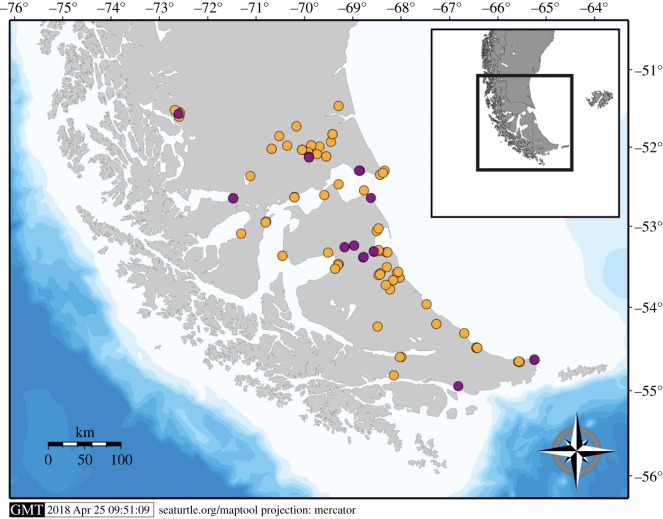
Map with pedestrian occupations. Purple points are misclassified by the algorithm as nautical, even though they are pedestrian, orange points are classified in coherence with the archaeological literature.

Aggregation areas of pedestrian sites can be observed in the continent, in Última Esperanza Sound area and, especially, in the area of the volcanic field of Pali Aike and the Río Gallegos Basin (figure 4). Coastal locations are placed, also, throughout the Atlantic Ocean coast and the northern shore of the Magallanes Strait. In the case of IGTDF, an important concentration area is placed in the North of the Island, comprising the coasts of San Sebastián Bay and Inútil Bay. The rest of the sites are distributed in inland and coastal landscapes, including the Beagle Channel, Atlantic Façade and the Khami Lake. The presence of pedestrian sites in the Western and Southern Channels is really low, and they are associated with the initial occupation of the Beagle Channel region [51]. The existence of a common technological landscape involving the Continent and IGTDF is strongly related to the maintenance and development of technological practices related to the glacial period when the island was a portion of the continent, what enabled the arrival of human groups to the area.

### 4.2. Social interaction

#### 4.2.1. Misclassified cases

The third main outcome of the analyses conducted is related to the occupations misclassified by the random forest algorithm: up to 24 cases. These misclassified sites are entries that do not follow the general pattern identified by the classification algorithm, which leads the latter to misclassify them. This behaviour reveals entries that need to be studied carefully, to understand the particularities of each case. In overall terms, two main reasons account for these misclassifications: first, the low frequencies of technological materials retrieved from these sites and/or the scarcity of data provided by publications; this situation encompasses 50% (*n* = 12) of the misclassified occupations, which are distributed in many different areas. Consequently, this outcome is not linked to a specific time-frame or excavation/analysis method.

**Figure 5.**
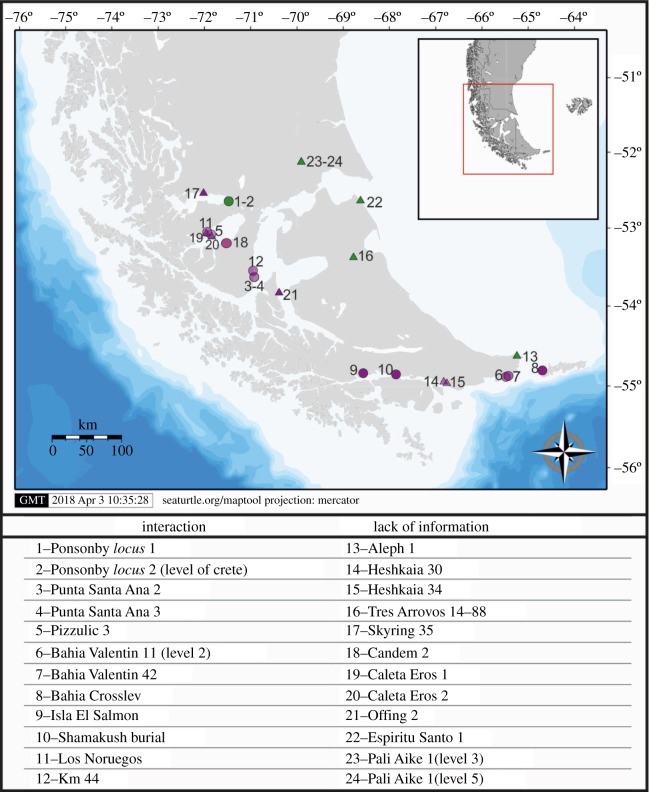
Misclassified points. Misclassified nautical points in purple, misclassified pedestrian points in green. Circles are misclassified points due to interaction. Triangles are misclassified points due to lack of information. In the table, 12 sites misclassified by the random forest classifier: we can see the name of the occupation, the classification of the site according to archaeological literature and the classification provided by the random forest algorithm.

Second, the existence of a group of misclassified sites which cannot be accurately classified into nautical or pedestrian, since in their records distinctive technological variables are scarce or absent and/or because they exhibit technological features from both nautical and pedestrian assemblages. Henceforth, these misclassified sites could be considered proxies of connectivity and interaction between nautical and pedestrian communities (figure 5). Most of the misclassified points in this second group are located in places of easy access between inland and marine landscapes, thus fostering communications among HG/HFG societies. Incidentally, the biggest aggregation of sites (*n* = 7) in this second group is scattered around the Otway Sound and the Magellan Strait, which has been traditionally indicated as an area of contact between nautical and pedestrian societies by ethnographical sources [32]. The same occurs with the assemblages of Mitre Peninsula (*n* = 2). In the case of Beagle Channel (*n* = 2), even though it has been considered a territory of nautical populations, the establishment of connections with Otway and Brunswick Peninsula is well documented by ethnography and ethnohistory [18,21,52].

In a nutshell, these results highlight the existence of networks of technological knowledge related to mobility patterns. This technological knowledge was transmitted and exchanged among the members of each society and between societies, particularly interesting being the interaction points between the two mobility trends, i.e. the sites where both nautical and pedestrian technologies can be found, which leads the algorithm to misclassify them.

## 5. Conclusion

The present paper provides relevant insights to understand the relationships between shared technology and mobility patterns in pedestrian and nautical HG/HFG societies.

A formal approach has been used to detect patterns of shared technology and to identify the variables which are more relevant to discriminate mobility. In this sense, we draw on a comprehensive database, which encompasses a wide range of technical features considered as material proxies, to identify interactions and social boundaries.

The analyses presented here demonstrate, first, that there is a strong and non-trivial dependence between technology and mobility patterns, the relation being based on a restricted number of technological items. This result suggests that the study area was highly integrated through the circulation of knowledge and/or a variety of materials, since most of the technological evidence is shared by all the HG/HFG societies in the region. But, at the same time, it highlights that these networks implied a heterogeneous circulation of technological knowledge among groups, in terms of manufacturing techniques, material objects and their geographical distribution. Furthermore, at a higher level of abstraction, the outcomes of group variable importance acknowledge which are the most significant proxies to distinguish mobility strategies, thus providing a hierarchy and broadening the spectrum of technological variables that could be explored to assess people connectedness.

In this sense, the methodological approach adopted here undercuts basic assumptions supported by archaeologists, regarding the identification of exceptional diagnostic features to establish social ties or boundaries between groups. Conversely, we propose to analyse technology ‘in action’, which involves a deep comprehension of how tools are produced and used by individuals and societies. The predictive models obtained using comprehensive technological assemblages improve the accuracy beyond that of direct classification based on decision trees, ‘index fossils’ or decorative styles. Therefore, and in accordance with the results provided by the group variable analysis conducted, we can emphasize the importance of the processes underlying the production of artefacts as reliable archaeological markers of both technological development and social interaction. An approach based on the technological processes needed to obtain the artefacts enables to overcome two common arguments in archaeological research related to the difficulty in establishing connectedness between groups based on the final lithic artefacts: (i) that their design responds to functional requirements and (ii) that there are certain morphologies which are distributed worldwide. It is highly recommended that future research devotes special attention to the processes underlying the production of artefacts as opposed to the shape of the artefact, which in accordance with our findings may not be the most distinctive trait.

Secondly, the classifiers implemented allow to identify the geographical areas where significant social interactions could have taken place (e.g. Otway–Skyring Sounds) as well as to distinguish regions with rather weak connections. This information, coming from the misclassified archaeological occupations, has been used to analyse in detail potential salient regions, detecting, in some cases, interaction areas or places with the absence of evidence. Further research will be addressed to assess the existence of geographical or social barriers which could have hindered the spread of technological knowledge.

The example of the present case study stresses the potential of exploring standardized and formalized broad-spectrum databases with advanced machine learning and statistical tools, since the models and conclusions obtained can serve as decision support tools and as analysis guide, not just for the present case, but the whole archaeological discipline.

## Supplementary Material

Binary Database of Patagonian Archaeological Technology

## Data accessibility

Bibliography and reports used to generate original database are available in: https://www.researchgate.net/publication/324217985_Reference_Database_of_Hunter-Gatherer_Technology_from_the_uttermost_tip_of_Patagonia_South_America (doi:10.13140/RG.2.2.11121.79202). Specific database used in the statistical analysis is included in the paper as electronic supplementary material.
